# Draft Genome Sequence of *Bacillus* sp. Strain EB106-08-02-XG196, Isolated from High-Nitrate-Contaminated Sediment

**DOI:** 10.1128/MRA.01149-20

**Published:** 2020-10-29

**Authors:** Xiaoxuan Ge, Michael P. Thorgersen, Farris L. Poole, Adam M. Deutschbauer, John-Marc Chandonia, Pavel S. Novichkov, Paul D. Adams, Adam P. Arkin, Terry C. Hazen, Michael W. W. Adams

**Affiliations:** a Department of Biochemistry and Molecular Biology, University of Georgia, Athens, Georgia, USA; b Environmental Genomics and Systems Biology Division, Lawrence Berkeley National Laboratory, Berkeley, California, USA; c Molecular Biosciences and Integrated Bioimaging, Lawrence Berkeley National Laboratory, Berkeley, California, USA; d Department of Bioengineering, University of California, Berkeley, Berkeley, California, USA; e Department of Civil and Environmental Engineering, University of Tennessee, Knoxville, Tennessee, USA; University of Rochester School of Medicine and Dentistry

## Abstract

*Bacillus* sp. strain EB106-08-02-XG196 was isolated from a high-nitrate- and heavy metal-contaminated site at the Oak Ridge Reservation in Tennessee. We report the draft genome sequence of this strain to provide insights into the genomic basis for surviving in this unique environment.

## Abstract (precis)

*Bacillus* sp. strain EB106-08-02-XG196 was isolated from a high-nitrate- and heavy metal-contaminated site at the Oak Ridge Reservation. We report the draft genome sequence of this strain to provide insights into the genomic basis for surviving in this unique environment.

## ANNOUNCEMENT

The Oak Ridge Reservation (ORR) in Tennessee contains a site termed the S-3 ponds, which has been used for the disposal of waste liquids produced by the Y-12 nuclear plant and is contaminated with nitrate and heavy metals (1). The groundwater at this site has a pH value as low as 3.0 and contains up to 230 mM nitrate and high concentrations of over 20 different metals (2–4). In contrast, the concentration of the metal molybdenum, which is essential for nitrate reduction by microorganisms, is lower in highly contaminated groundwater than it is in the uncontaminated pristine groundwater (5, 6).

Strain EB106-08-02-XG196 (referred to as XG196) was isolated from highly contaminated ORR sediment core EB106, located 21 m downstream of the S-3 ponds (5). It was identified as a *Bacillus* sp. strain by 16S rRNA gene sequencing. The ORR sediment sample was collected and processed under anaerobic conditions (5). Sediment samples (1 g) were incubated anaerobically in 5 ml of a defined medium described previously (7). Mixed carbon sources (formate, acetate, ethanol, lactate, succinate, and glucose, 2 mM each) and 0.1 g/liter yeast extract were used for the enrichment. Cultures were incubated at room temperature for 2 to 7 days and then streaked onto agar plates of defined medium. A single colony of strain XG196 was isolated and cultivated for genomic DNA preparation.

XG196 genomic DNA was prepared using a ZymoBead genomic DNA kit (Zymo Research, Irvine, CA, USA). The Illumina sequencing library was made by shearing DNA to ∼800 bp (Covaris, Woburn, MA, USA), and the KAPA HyperPrep kit (Roche, Basel, Switzerland) was then used to add Illumina-compatible adapters to the library. The genome was sequenced using the Illumina HiSeq 2500 platform. This generated 10,798,254 paired-end reads of 151 bp each, totaling 1,630,536,354 bp, with a genome coverage of 215× and an *N*_50_ value of 283,772 bp. The sequencing reads were trimmed using Trimmomatic v0.36 (8) and assembled *de novo* using SPAdes v3.12.0 (9). An initial genome annotation was performed using Prokka v1.12 (10), executed using KBase (11). The KBase applications kb_trimmomatic v1.2.13, kb_SPAdes v1.1.3, and ProkkaAnnotation v2.0.3 were used for the corresponding steps in the pipeline. Default parameters were used for all software unless otherwise specified. Species trees were built for the XG196 genome using two KBase software packages, GTDB-Tk Classify v1.3.0 and Insert Genome Into Species Tree v2.1.10. Both rely on phylogenetic marker genes other than 16S rRNA, and both identified Bacillus niacini as having the genome most similar to that of XG196 (11). Finally, the KBase-derived assembly was deposited in GenBank. Due to compatibility issues, the GenBank PGAP was rerun on the assembly to produce the final draft genome. We ran the RAST annotation pipeline in KBase v0.1.1 on the GenBank-derived genome to classify features. The final draft genome of strain XG196 contained 6,010,169 bp in 55 contigs longer than 500 bp, with a G+C content of 38.35%. A total of 5,721 coding genes and 234 noncoding features were annotated, including 78 noncoding RNA genes, 42 noncoding regulatory features, and 114 other noncoding features.

### Data availability.

This whole-genome shotgun project has been deposited in GenBank under the accession number JABWSY000000000. The version described in this paper is the first version (JABWSY000000000.1). The raw sequence reads have been deposited in the SRA under the accession number PRJNA633127. All KBase analyses and data objects are available in the KBase static narrative (https://kbase.us/n/60201/86).
